# A structural role for tryptophan in proteins, and the ubiquitous Trp C^δ1^—H⋯O=C (backbone) hydrogen bond

**DOI:** 10.1107/S2059798324005515

**Published:** 2024-06-28

**Authors:** Michal Szczygiel, Urszula Derewenda, Steve Scheiner, Wladek Minor, Zygmunt S. Derewenda

**Affiliations:** a Department of Molecular Physiology and Biological Physics University of Virginia 1340 Jefferson Park Avenue Charlottesville VA22908-0736 USA; b Department of Chemistry and Biochemistry Utah State University Logan Utah USA; National Hellenic Research Foundation, Greece

**Keywords:** tryptophan, hydrogen bonds, C—H⋯O bonds, protein structure

## Abstract

Tryptophan is the most prominent amino acid found in proteins, with multiple functional roles. Its side chain is made up of the hydrophobic indole moiety, with two groups that act as donors in hydrogen bonds: the N^ɛ^—H group, which is a potent donor in canonical hydrogen bonds, and a polarized C^δ1^—H group, which is capable of forming weaker, noncanonical hydrogen bonds. Due to adjacent electron-withdrawing moieties, C—H⋯O hydrogen bonds are ubiquitous in macromolecules, albeit contingent on the polarization of the donor C—H group. Consequently, C^α^—H groups (adjacent to the carbonyl and amino groups of flanking peptide bonds), as well as the C^ɛ1^—H and C^δ2^—H groups of histidines (adjacent to imidazole N atoms), are known to serve as donors in hydrogen bonds, for example stabilizing parallel and antiparallel β-sheets. However, the nature and the functional role of interactions involving the C^δ1^—H group of the indole ring of tryptophan are not well characterized. Here, data mining of high-resolution (*r* ≤ 1.5 Å) crystal structures from the Protein Data Bank was performed and ubiquitous close contacts between the C^δ1^—H groups of tryptophan and a range of electronegative acceptors were identified, specifically main-chain carbonyl O atoms immediately upstream and downstream in the polypeptide chain. The stereochemical analysis shows that most of the interactions bear all of the hallmarks of proper hydrogen bonds. At the same time, their cohesive nature is confirmed by quantum-chemical calculations, which reveal interaction energies of 1.5–3.0 kcal mol^−1^, depending on the specific stereochemistry.

## Abstract 2

A set of hydrogen bonds which occur in proteins between the C^δ1^—H donor group of the tryptophan indole and main-chain carbonyl O atoms separated by 1–4 peptide units were characterized. These interactions stabilize unique and functionally important structural motifs and noncanonical side-chain conformations of tryptophan.

## 1. Introduction

Tryptophan (Trp) is the largest amino acid, with important functional roles in proteins. It is often found at protein–protein interfaces, such as antibody–antigen interfaces, accounting for tight interactions and specificity (Samanta & Chakrabarti, 2001 ▸), and is ubiquitous in the ligand/substrate-binding sites of, for example, lectins and various enzymes (Zhang *et al.*, 2004 ▸; Spier & Lummis, 2000 ▸). It is also enriched on the surface of membrane proteins embedded in the lipid membrane, where its hydrophobic indole moiety interacts intimately with the lipid phase (Khemaissa *et al.*, 2021 ▸). The multiple functions of Trp are contingent on the conformation that it adopts in the active site or at the interface. Consequently, understanding the nature of the forces stabilizing the discrete conformations of this amino acid is essential in structural biology and drug discovery.

**Figure 1:**
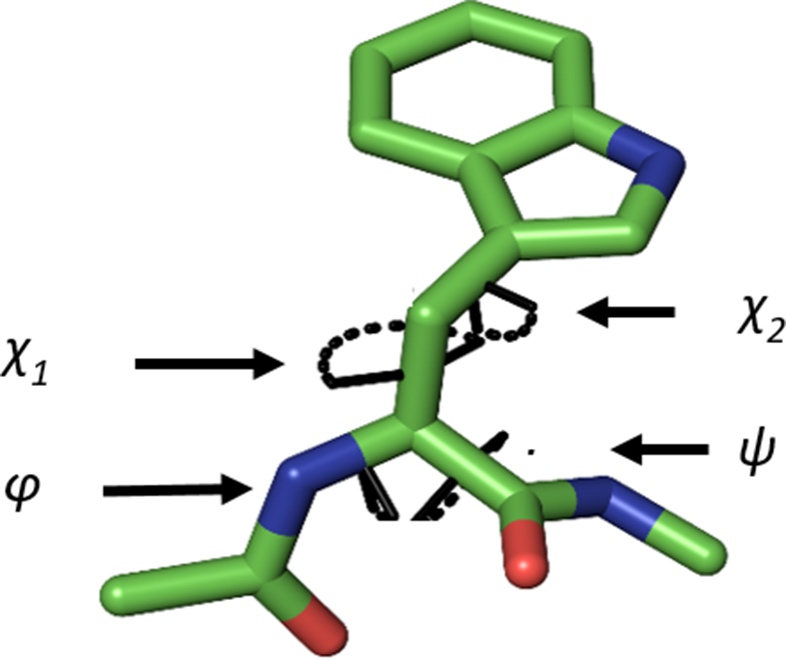
The four conformational dihedral angles defining the structure of a tryptophan residue within a polypeptide.

The structure of Trp is defined by four dihedral angles (Fig. 1 ▸): the backbone Ramachandran φ and ψ angles and the two side-chain dihedral angles χ_1_ and χ_2_. The first, χ_1_, is a rotameric angle with minimum energies at −60° (*g*−, or **m**), +60° (*g*+, or **p**) and 180° (*trans*, or **t**). In contrast, χ_2_, which involves the *sp*^2^ γ-carbon, should in theory only assume values of −90° or +90°. However, it was noted early on that a significant cohort of Trp residues in proteins exhibit an unfavourable **m**0 conformation (Lovell *et al.*, 2000 ▸; we follow the notation introduced by Lovell and coworkers here, where the letter **m**, **p** and **t** is followed by the value of the χ_2_ angle). Recent results reaffirm that **m**0 constitutes ∼10% of the Trp conformers in proteins, while **m**95 and **t**-105 dominate the conformational space, with a combined frequency of 65.7% (Hameduh *et al.*, 2023 ▸). The question that arises is what are the noncovalent interactions that are responsible for stabilizing the conformations of Trp, especially noncanonical conformations. In the first attempt to address this question, Petrella & Karplus (2004 ▸) studied 25 protein crystal structures determined at a resolution of 2.0 Å or higher. Based on observed stereochemistry and molecular-dynamics calculations, they concluded that C—H⋯O hydrogen bonds, including those with ^Trp^C^δ1^—H as a donor, were involved in stabilizing the **m**0 conformation. In contrast, a subsequent more comprehensive study of nonredundant protein crystal structures determined to better than 2.5 Å resolution concluded that the C^δ1^—H group does not appear to impact the local stereochemistry, perhaps due to a low energy of the interactions (Nanda & Schmiedekamp, 2008 ▸).

The existence of hydrogen bonds in which a polarized C—H group can serve as a donor was initially invoked in 1937 to explain the physical properties of mixtures of chloroform with acetone (Glasstone, 1937 ▸). Subsequently, such hydrogen bonds have been independently postulated based on the stereochemistry of selected intermolecular interactions observed in the crystal structures of organic compounds (Sutor, 1962 ▸, 1963 ▸; Taylor & Kennard, 1982 ▸). More recent spectroscopic (for example infrared and NMR) and computational studies provided detailed insights into the nature of this class of interactions (Hobza & Havlas, 2000 ▸; Joseph & Jemmis, 2007 ▸; Majerz & Olovsson, 2012 ▸; Driver *et al.*, 2016 ▸; Shi & Min, 2023 ▸; Gilli *et al.*, 1994 ▸; Gilli & Gilli, 2000 ▸; Isaacs *et al.*, 1999 ▸, 2000 ▸; Derewenda, 2023 ▸). As a result, the current definition of a hydrogen bond endorsed by IUPAC includes C—H groups as donors (Arunan *et al.*, 2011 ▸).

Although generally regarded as being significantly weaker than canonical hydrogen bonds, the interaction energy of C—H⋯O bonds is enhanced if the C—H group is polarized by an adjacent electron-withdrawing moiety, such as nitrogen in heterocyclic compounds next to a methine group, *i.e.* =CH—. Biological macromolecules, *i.e.* proteins and nucleic acids, contain a number of such groups capable of forming C—H⋯O bonds. The occurrence and significance of these interactions have been the subject of several comprehensive reviews (Scheiner, 2006*a* ▸; Gu *et al.*, 1999 ▸; Horowitz & Trievel, 2012 ▸; Derewenda, 2023 ▸). In DNA and RNA, methine groups in nitrogen bases are involved in base-pairing and base–pentose interactions (Beiranvand *et al.*, 2021 ▸; Yurenko *et al.*, 2011 ▸; Balaceanu *et al.*, 2017 ▸). In proteins, the side chain of histidine contains highly polarized C^ɛ1^—H and C^δ2^—H groups (particularly in the protonated, *i.e.* imidazolium, state) which are often involved in hydrogen bonds (Steinert *et al.*, 2022 ▸), including functionally important groups in the active sites of enzymes such as serine hydrolases (Derewenda *et al.*, 1994 ▸). The main-chain C^α^—H group is another example of a polarized bond, despite the *sp*^3^ hybridization of carbon, owing to the adjacent electron-withdrawing peptide linkages. These groups are directly involved in stabilizing the β secondary structure via C^α^—H⋯O=C interstrand bonds in both parallel and antiparallel sheets (Derewenda *et al.*, 1995 ▸; Scheiner, 2005 ▸, 2006*b* ▸, 2010 ▸).

Tryptophan contains a polarized methine group within the indole moiety. The N^ɛ1^ atom polarizes the adjacent C^δ1^—H bond, making it suitable to serve as a hydrogen-bond donor. *Ab initio* calculations showed the energy of such a hydrogen bond to a water molecule to be −2.1 kcal mol^−1^, with a C⋯O distance of 3.35 Å (Scheiner *et al.*, 2002 ▸). Given the dramatic increase in the number of protein structures determined at high resolution, particularly during the Structural Genomics Initiative (Standley *et al.*, 2022 ▸), we decided to revisit the question of the role of the ^Trp^C^δ1^—H group in protein structures and its possible role in Trp side-chain stereochemistry. Using a subset of nonredundant protein structures from the PDB, with a conservative resolution cutoff of 1.5 Å, we discovered that C^δ1^—H groups have a high propensity to interact with main-chain carbonyl O atoms, specifically with those located nearby in the polypeptide chain. Our stereochemical analysis is consistent with the notion that these interactions have all of the properties of hydrogen bonds, and quantum-mechanical calculations of interaction energies corroborate this conclusion. The presence of hydrogen bonds involving the ^Trp^C^δ1^—H group correlates with hitherto uncharacterized discrete structural motifs, with important implications for protein structure and function.

## 2. Methods

### 2.1. Data mining in the Protein Data Bank and stereochemical analysis

A subset of crystal structures determined to a resolution of 1.5 Å or better was extracted from the Protein Data Bank. Redundancy was reduced by using a maximum 95% amino-acid identity cutoff. This resulted in a database of 7911 structures. The vast majority did not contain H atoms; those that did had various C—H distances depending on the refinement program used. Notably, *Phenix* uses 0.93 Å, which is significantly shorter than the actual value of the C^δ1^—H distance in indole/tryptophan. It is well established from spectroscopy that the C—H distance shortens in the ethane/ethene/ethyne series, from 1.099 to 1.091 and 1.070 Å, respectively, although the difference may not stem from hybridization but from the coordination number of carbon (Vermeeren *et al.*, 2021 ▸). Inspection of the crystal structures of multiple Trp derivatives in the Cambridge Structural Database shows a variation from 0.93 to 1.13 Å, a range of ∼20% (data not shown). The most accurate measurements of the C—H bonds in crystals are from neutron diffraction. They show that *sp*^3^ and *sp*^2^ C—H bonds shorten to 1.092 and 1.081 Å, respectively (Lu *et al.*, 2021 ▸). As *PyMOL* adds riding H atoms to C^δ1^ of Trp at 1.09 Å, we used its algorithm to add them to all investigated structures, thus replacing the existing atoms.

This database was searched for any contacts between the H atoms of the C^δ1^—H and O atoms, with a *d*_HO_ distance of 2.86 Å (sum of van der Walls radii) and a minimum α_H_ of 110° (recommended as a minimum hydrogen-bond angle by IUPAC). In this study, we relied on a set of van der Waals radii that differ from those introduced by Bondi (1964 ▸), which are still routinely used. A recent reassessment of the atomic values of van der Waals radii (Chernyshov *et al.*, 2020 ▸) noted that Bondi’s values consistently underestimate the position of the energy minima by 0.3–0.4 Å. Using a new concept of line-of-sight and also taking chemical context into account, Chernyshov *et al.* (2020 ▸) provided a revised set of values. They suggest values of 1.21 Å for hydrogen in the context of C—H⋯*X* contacts (where *X* is not hydrogen) and 1.65 Å for an *sp*^2^ oxygen in a neutral carbonyl group. The sum, 2.86 Å, is the value we use rather than 2.72 Å, which would reflect Bondi’s values. Similarly, we note that the new sum of van der Waals radii for *sp*^2^ carbon and *sp*^2^ carbonyl oxygen is 3.56 Å rather than 3.22 Å, as previously inferred from Bondi’s values. Importantly, the estimate of 3.56 Å is more in line with the observed C⋯O distances in C—H⋯O bonds, established theoretically as 3.35 Å for ^Trp^C^δ1^—H⋯water (Scheiner *et al.*, 2002 ▸) and experimentally as 3.34 Å between methine in theophylline and oxygen in formaldehyde (Southern & Bryce, 2022 ▸).

The resulting database of close contacts had another layer of redundancy due to the presence of noncrystallographic symmetry, which includes biologically relevant oligomers. To eliminate multiple observations of the same contact, we arbitrarily selected the median interaction from oligomeric structures. We assumed that at 1.5 Å resolution or higher, differences between monomers may be due to genuine differences in crystal packing, and so averaging would not be appropriate. However, as the shortest distances might be encumbered by errors, the median contact might be more representative. This final nonredundant data set was used for further calculations of stereochemistry.

The stereochemical analysis was also performed using the *PyMOL* scripting engine. For each contact identified, the exact distance between the H atom and the O atom was determined, as well as additional geometric parameters as described in Section 3. The database was then split into clusters depending on the number of amino acids between the donor and acceptor groups. The data arising were recorded in tabular form using *Excel* for each identified conformational cluster separately. All statistical analysis was then carried out in *Excel*.

### 2.2. Quantum-chemical calculations of interaction energies

Quantum-chemical calculations were performed via the density-functional approach (DFT) within the context of the M06-2X functional (Zhao & Truhlar, 2008 ▸), which has been shown to be an accurate means of treating hydrogen bonds and related noncovalent bonds (Kříž & Řezáč, 2022 ▸; Boese, 2015 ▸; Kozuch & Martin, 2013 ▸; Walker *et al.*, 2013 ▸; Thanthiriwatte *et al.*, 2011 ▸; Liao *et al.*, 2003 ▸; Deible *et al.*, 2014 ▸; Li *et al.*, 2014 ▸; Mardirossian & Head-Gordon, 2013 ▸; Elm *et al.*, 2013 ▸; Bhattacharyya *et al.*, 2013 ▸). A polarized triple-ζ def2-TZVP basis set was chosen so as to afford a large and flexible set. The *Gaussian* 16 program (Frisch *et al.*, 2016 ▸) was chosen as the specific means to conduct these computations. The interaction energy *E*_int_ of each dyad was evaluated as the difference between the energy of the complex and the sum of the energies of the two constituent subunits. The counterpoise procedure (Boys & Bernardi, 1970 ▸) was applied to correct basis-set superposition error.

## 3. Results and discussion

### 3.1. Identification of interactions involving ^Trp^C^δ1^—H as the donor group

We generated a database of nonredundant protein crystal structures refined at a resolution of 1.5 Å or higher from the Protein Data Bank (Burley *et al.*, 2022 ▸; see Section 2 for the definition of redundancy *etc.*). Next, we calculated the positions of riding H atoms in all structures with the ^Trp^C^δ1^—H distance set to 1.09 Å. We then identified interactions involving ^Trp^C^δ1^—H groups as donors and potential oxygen acceptors, *i.e.* waters, hydroxyl groups (Ser, Thr and Tyr), side-chain groups (Asx and Glx) and main-chain carbonyl O atoms, using a maximum distance cutoff for H⋯O (*d*_HO_) of 2.86 Å and a minimum C^δ1^—H⋯O angle (α_H_) of 110° (see Section 2 for an explanation of the cutoff criteria).

We obtained 17 012 close contacts, 5983 of which were with water O atoms. Another 1046 contacts involved Glu and Asp carboxylate groups and 1010 contacts were with side-chain hydroxyl groups of Ser, Thr and Tyr. A further 542 contacts involved side-chain carbonyl groups of Asn and Gln. Interestingly, nearly half of all contacts, *i.e.* 8431 (49.6%), were with backbone carbonyl O atoms, which are particularly strong acceptors owing to their partial negative charge. Given the preponderance of these interactions, we focused on this group of contacts and analysed the respective stereochemistry in order to assess their character and potential function.

### 3.2. The stereochemistry of the ^Trp^C^δ1^—H⋯O=C^backbone^ contacts

**Figure 2:**
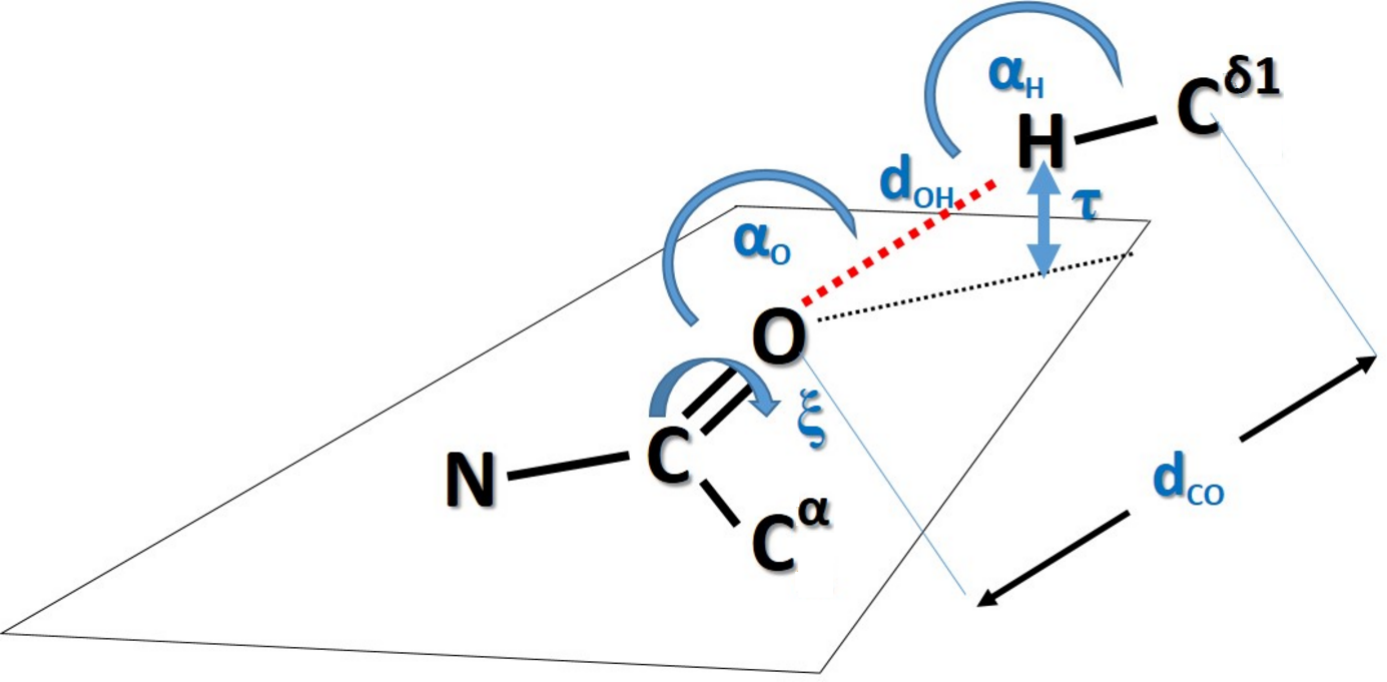
The stereochemical parameters used in this study. *d*_HO_, *d*_CO_ and τ are given in ångströms and all angles are given in degrees.

In order to characterize the stereochemistry of interactions involving ^Trp^C^δ1^—H groups, we first calculated the distribution of the donor–acceptor, or C⋯O, distances (*d*_CO_), as well as the C—H⋯O angles (α_H_), separately for all carbonyl O atoms as donors and for water O atoms (Figs. 2 ▸ and 3 ▸). The distribution of distances to carbonyl O atoms has a distinct maximum at 3.35 Å. In contrast, water O atoms were found further away on average, at 3.55 Å. The shortest distances in both cases were just below 3 Å. α_H_ increases gradually for both types of interactions with the C⋯O distance.

It should be stressed that intramolecular steric constraints significantly impact the observed distance distributions. Nevertheless, we note interesting trends. The peak of the *d*_HO_ distribution is shorter by 0.2 Å compared with the sum of the van der Waals radii of O and C atoms used in this study (*i.e.* 3.56 Å; see Section 2), suggesting a cohesive interaction. The higher deviation from linearity than observed in canonical hydrogen bonds can be rationalized in terms of the van der Waals interactions between the donor C and acceptor O atom. Specifically, at shorter C⋯O distances the α_H_ angle assumes more acute values, as the H atom is pushed out to avoid steric collision between H and O, which are further apart by at least 0.3 Å than the corresponding distance in canonical hydrogen bonds, owing to the partly covalent character of the latter. Overall, the stereochemistry is consistent with that expected for C—H⋯O hydrogen bonds in small organic molecules (Taylor & Kennard, 1982 ▸).

**Figure 4:**
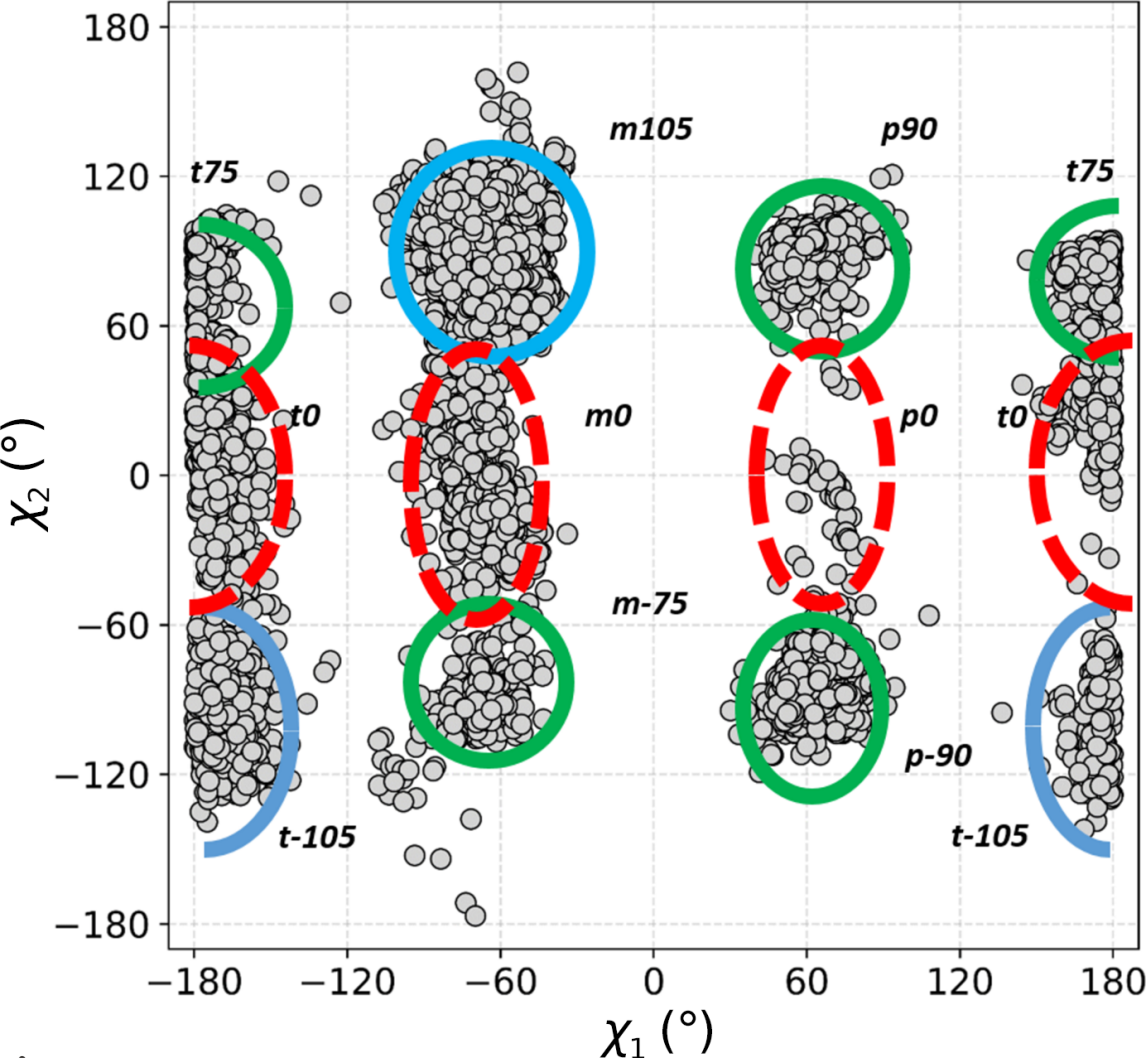
Distribution of side-chain dihedral angles for Trp residues involved in contacts with all main-chain carbonyl O atoms. Blue outlines indicate the most populous, low-energy clusters found in proteins, green shows energetically favourable but less common clusters and red represents theoretically unfavourable conformations.

Next, we calculated a scatter plot of the two Trp side-chain dihedral angles, *i.e.* χ_1_ and χ_2_, for all ^Trp^C^δ1^—H⋯O=C^backbone^ contacts (Fig. 4 ▸). The purpose was to investigate whether the various structural motifs involve Trp side chains in canonical or strained conformations. Nine conformer clusters are observed. The results are intriguing: although low-energy **m**105 and **t**-105 are the dominant clusters, as expected, not only is **m**0 strongly represented, but the unfavourable **t**0 has a nearly equal frequency, and some cases of **p**0 are also identifiable.

**Figure 5:**
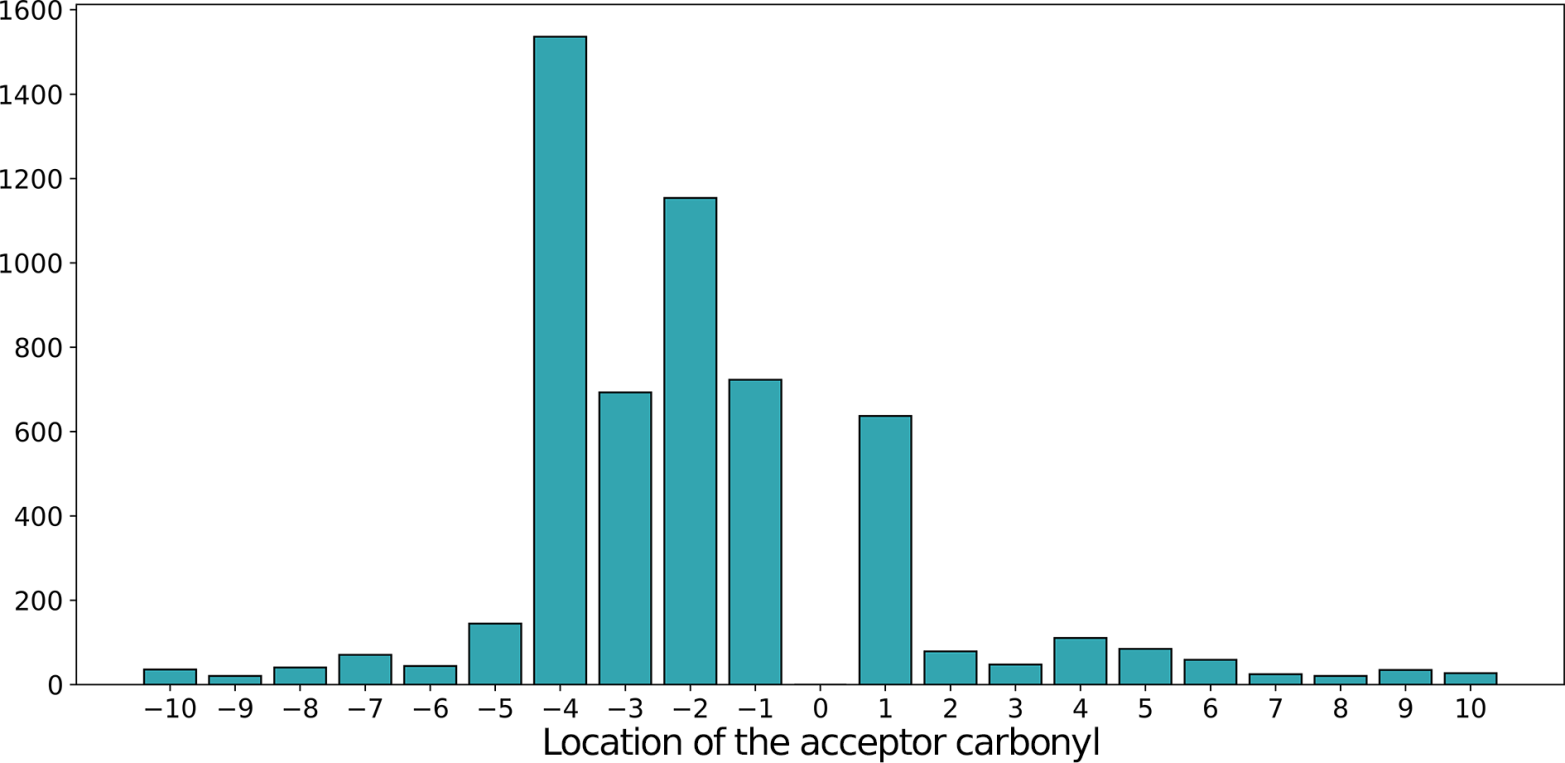
A histogram showing the number of contacts between ^Trp^C^δ1^—H as a donor and the *i*th main-chain carbonyl O atom as the acceptor. For example, −2 denotes an acceptor located two peptide units upstream in the sequence.

We then asked what the separation was for the observed pairs of interacting moieties along the polypeptide chain. Fig. 5 ▸ illustrates the relative register in the sequence between the donor Trp and the acceptor carbonyl group. Positive values indicate that acceptor O atoms are located downstream in the sequence, and negative values refer to oxygen acceptors that are located upstream, *i.e.* towards the amino-terminus. The most common interactions are those with peptide O atoms in nearby positions: +1, −1, −2, −3 and −4 (Fig. 5 ▸). Intrigued by this observation, we carried out additional stereochemical characterization for all contacts within each class (Fig. 2 ▸), including the C=O⋯H angle (α_O_) and the C^α^—C=O⋯H dihedral angle (ξ), which allowed calculation of the elevation of the hydrogen from the *sp*^2^ plane (τ). Canonical hydrogen bonds demonstrate a strong preference for hydrogens to cluster with α_O_ angles in the range 120–240° and close to the *sp*^2^ plane (*i.e.* low elevation; Murray-Rust & Glusker, 1984 ▸), and similar trends, albeit not as pronounced, have been reported for C—H⋯O bonds (Taylor & Kennard, 1982 ▸). We were interested in whether we could reproduce these trends in the present study. Finally, we calculated the Ramachandran angles for all of the Trp residues involved to identify possible correlations between local secondary structure and side-chain conformation.

All calculations up to this point were carried out using raw coordinates from the Protein Data Bank (except for the riding hydrogen positions, which were added independently). As we embarked on the detailed analysis of specific structures, we were concerned about inconsistencies inherent in the data sets in the PDB introduced by different protocols or refinement and different software. Specifically, we were concerned about the lack of inclusion of H atoms during refinement, the lack of coordinates in the file *etc*. To avoid bias, all structures described below were subjected to additional standardized refinement and addition of riding H atoms at correct, uniform positions using the *PyMOL* script. Details are described in the supporting information and Supplementary Table S1.

#### 3.2.1. The C^δ1^—H → O=C (+1) class

**Figure 6:**
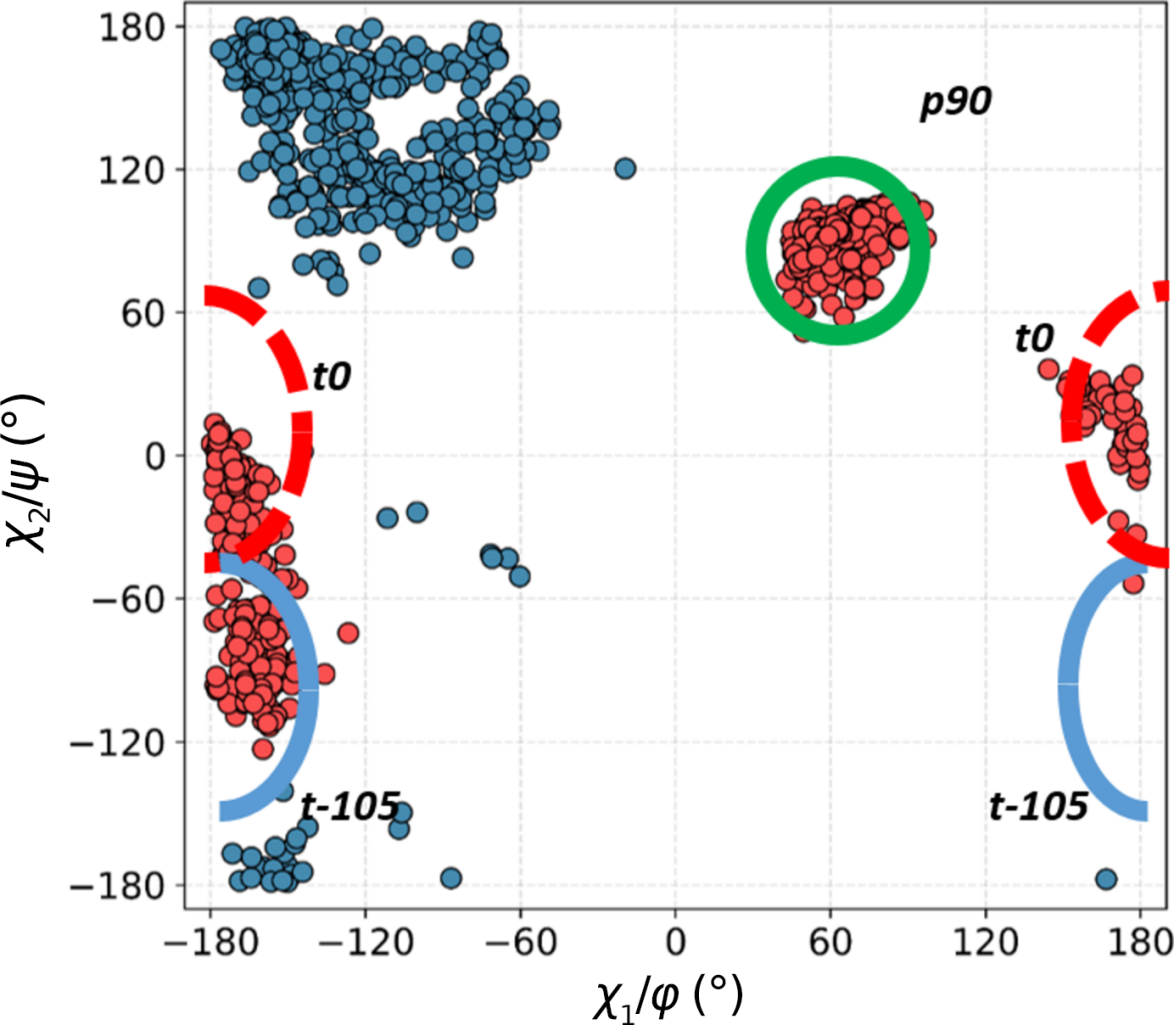
A double scatter plot (Ramachandran φ/ψ, blue; conformational, χ_1_/χ_2_, red) for Trp residues in all structural motifs in the +1 class. The clusters are identified by type as shown in Fig. 4 ▸.

**Figure 7:**
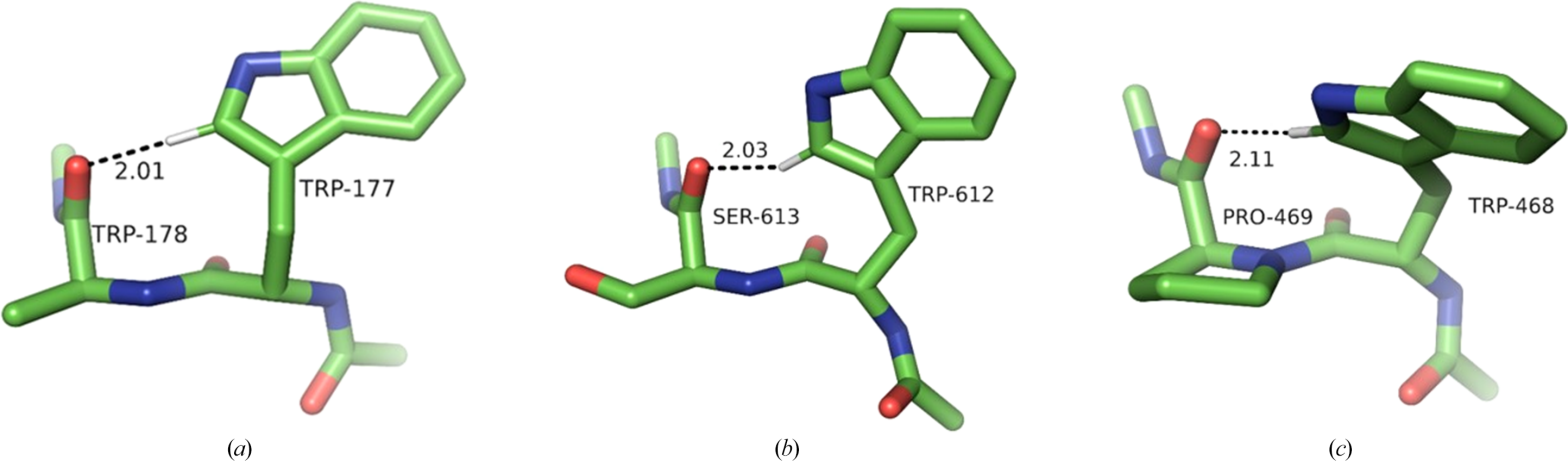
Examples of the three conformational Trp clusters in the +1 class. (*a*) **p**90 (PDB entry 1v5v; only the C^β^ atom of Trp178 is shown for clarity), (*b*) **t**0 (PDB entry 3ts3), (*c*) **t**-105 (PDB entry 4ge6).

In this class of interactions, the C^δ1^—H group of Trp points towards the carbonyl O atom of the next residue downstream in the sequence, reaching across a single peptide bond. This requires a favourable combination of four dihedral angles: two Ramachandran angles, ψ in Trp and φ in the residue downstream, and both the χ_1_ and χ_2_ angles in the Trp side chain. There are three possible combinations, leading to only three specific conformational clusters out of the nine possible (Fig. 6 ▸). The most populous (361 structures) is a distinct, tight cluster corresponding to the rather rare (4.7% frequency) **p**90 conformer (average χ_1_ and χ_2_ of 64° and 90°, respectively). The Trp residue is invariably in the β-secondary structure and the C^δ1^—H approaches the acceptor O atom from the *re* face. The bond is close to linear (the average α_H_ is 153°), but the angle on the acceptor is unfavourable (average α_O_ of 107°; Fig. 7 ▸*a*), resulting in the hydrogen being located significantly outside the *sp*^2^ plane of oxygen (average 2.2 Å). A number of such interactions result in very close *d*_HO_ distances.

Both remaining clusters are in the *trans* conformation with χ_1_ close to 180°. The first is identifiable as **t**0 (154 structures). In this cluster, C^δ1^—H also approaches the O atom from the *re* face (as defined by IUPAC), with H significantly out of the *sp*^2^ plane, and the *d*_HO_ distances are often short. The bond tends to be less linear than in **p**90, with an average α_H_ of 137°, and the angle on the acceptor (α_O_) is unfavourable (average of 106°) (Fig. 7 ▸*b*), although the H atom is closer to the *sp*^2^ plane (average τ of 1.9 Å).

The second *trans* cluster is the canonical **t**-105 (113 structures), showing optimal C^δ1^—H⋯O bond stereochemistry. This is accomplished specifically when the downstream residue is proline (15 of the 30 shortest distances, including the five shortest distances) or alanine (nine of the 30 shortest distances). The reason is that the secondary structure of this residue needs to be of the collagen type, and both proline and alanine have a strong preference for this conformation (Berisio *et al.*, 2002 ▸; Parchaňský *et al.*, 2013 ▸). The stereochemistry leads to a mean α_O_ of 123°, with the hydrogen on average only 0.6 Å out of the *sp*^2^ plane, in an excellent position to interact with one of the free *sp*^2^ electron pairs of oxygen (Fig. 7 ▸*c*).

#### 3.2.2. The C^δ1^—H → O=C (−1) class

**Figure 8:**
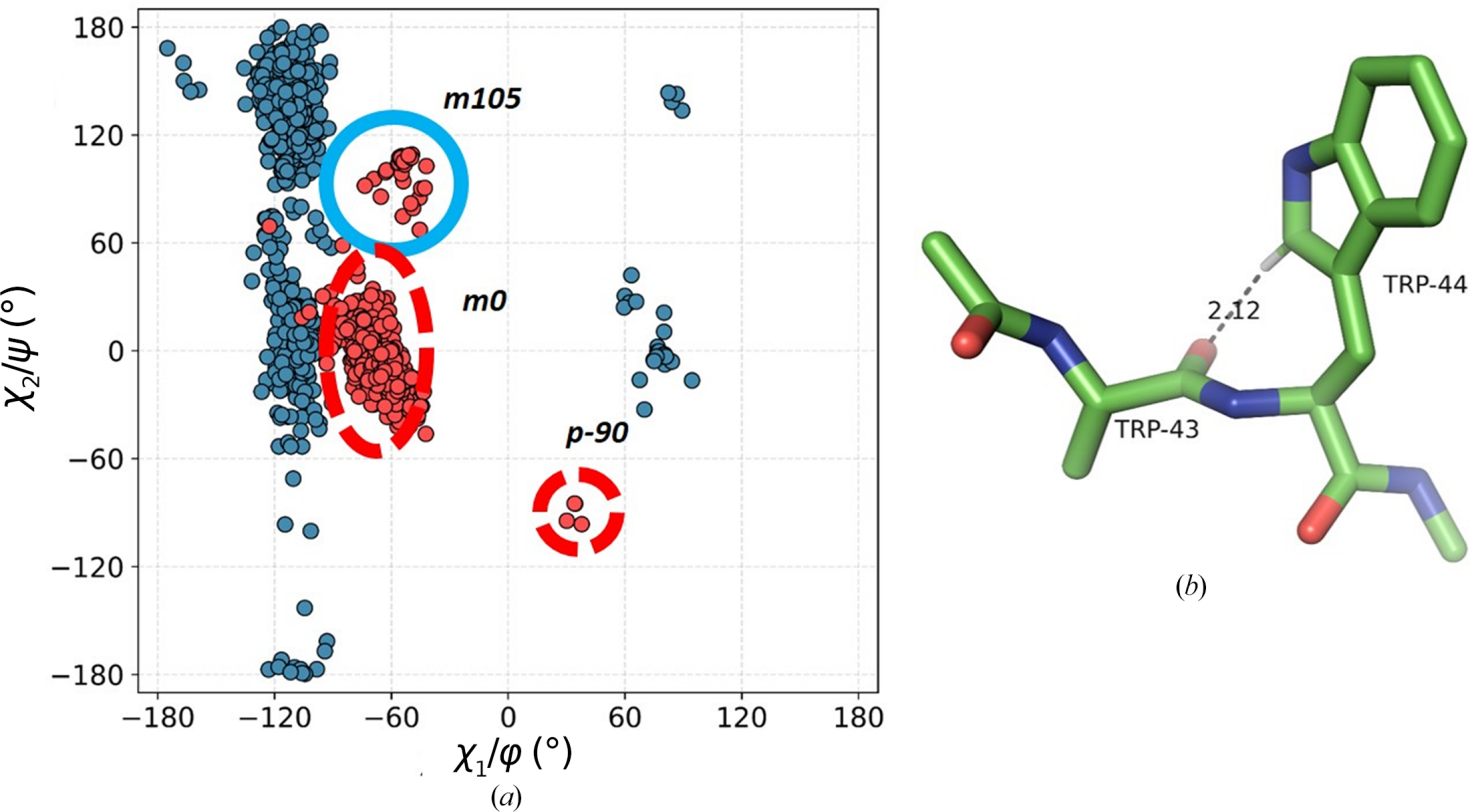
Class −1 of interactions. (*a*) A double scatter plot (Ramachandran φ/ψ, blue; conformational, χ_1_/χ_2_, red) for Trp residues in all motifs. (*b*) An example from the **m**0 cluster (PDB entry 2df6; only the C^β^ atom of Trp43 is shown for clarity).

In this unique type of contact, the C^δ1^—H group of the indole ring points towards the preceding peptide, engaging in an interaction with the carbonyl O atom immediately upstream in the sequence. The vast majority in this group (694 structures) are in the unfavourable **m**0 conformation, initially identified by Lovell *et al.* (2000 ▸). Our observation rationalizes the high frequency of this conformer. The motif restricts the Ramachandran φ angle to a narrow range of −90° to −135°, while ψ is allowed a broader range (Fig. 8 ▸). The average χ_2_ is −3.2°. Although the C—H⋯O interaction is close to linear (the average α_H_ is 146.3°), the average α_O_ is very unfavourable (86°) and the hydrogen is out of the amide plane by more than 2 Å on average. We note that such motifs often occur within a β-strand or at the end of one, resulting in a sharp turn.

A small minority of contacts in this class, *i.e.* 30 examples, are of the **m**105 type and almost all involve Trp residues in the α_L_ region of the Ramachandran plot, with long *d*_HO_ distances. Such stereochemistry suggests weak interactions. There are only three structures in the **p**-90 cluster.

#### 3.2.3. The C^δ1^—H → O=C (−2) class

**Figure 9:**
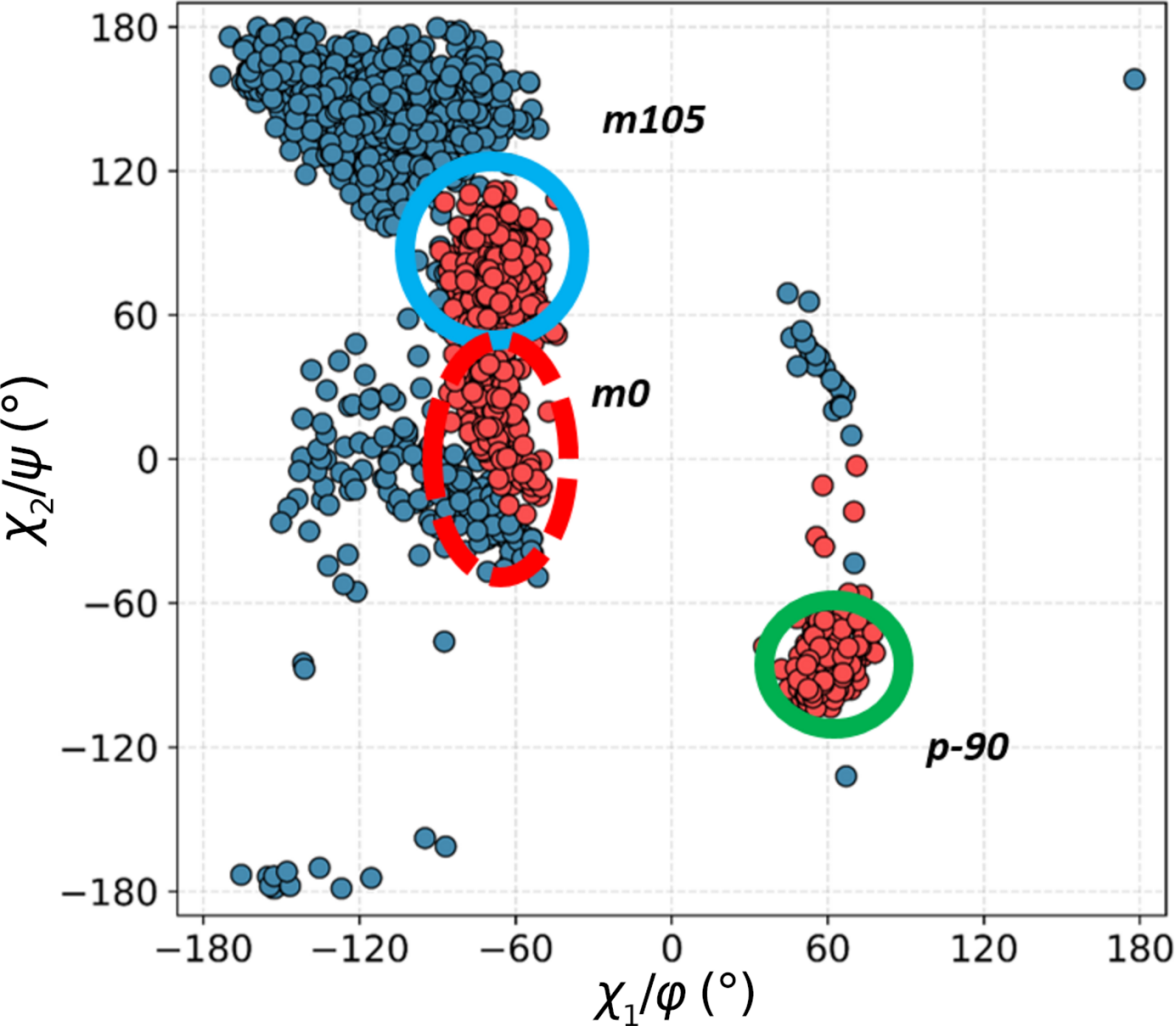
The −2 class of interactions. A double scatter plot (Ramachandran φ/ψ, blue; conformational, χ_1_/χ_2_, red) for Trp residues in all structural motifs identified in this class.

More conformational freedom is allowed in this class of contacts owing to the insertion of a residue between the acceptor and Trp. Although this motif is more diverse, the same three conformational clusters are observed as were seen in the previous class, albeit with very different frequencies (Fig. 9 ▸). By far the most common here is the canonical **m**105 conformation, with 698 structures. With very few exceptions, Trp is in the β-secondary conformation, with the hydrogen this time approaching from the *si* face and significantly outside the *sp*^2^ plane. The average α_H_ and α_O_ angles are 137° and 114°, respectively.

The **m**0 cluster is represented by 190 structures. It is very close in conformational space to **m**105 because the **m**105 structures are shifted to lower χ_2_, with an average value of 82°, while the **m**0 cluster is also shifted to higher values of χ_2_, with an average of 23°. In both groups Trp is primarily found in extended, β-secondary conformations, although right-handed and left-handed helical structures are also observed.

**Figure 10:**
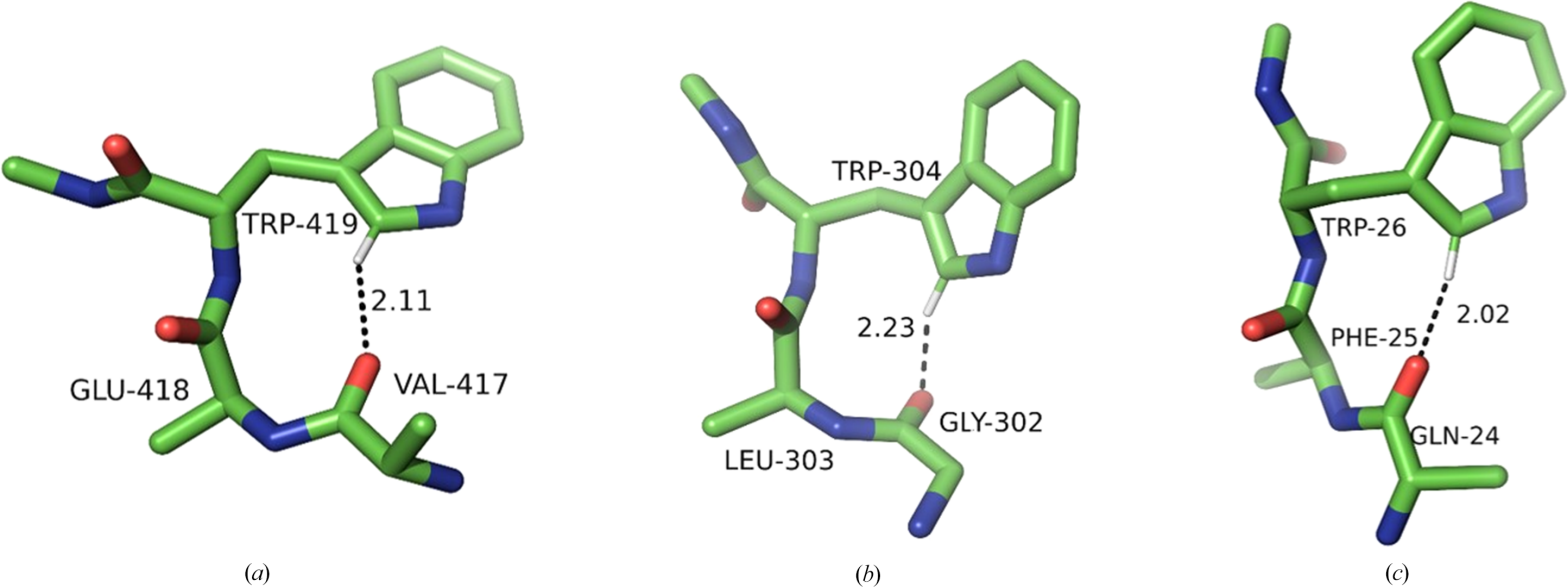
Examples of the three conformational Trp clusters in the +2 class. (*a*) **m**0 (PDB entry 5k4b; only the C^β^ atoms of non-Trp residues are shown for clarity), (*b*) **m**105 (PDB entry 2vbk), (*c*) **p**-90 (PDB entry 6qo9).

There are 277 motifs that constitute the **p**-90 cluster. The secondary conformation of Trp is restricted to right-handed α-helices and β-structure only. Examples of each of the clusters are shown in Fig. 10 ▸.

Of note is the fact that many of the motifs in all three clusters resemble the classic type II β-turn. The conformation of Trp is such that the C^δ1^—H group mimics the peptide amide which would serve as a donor in a classical β-turn, adding just one atom to the turn (11 atoms instead of 10). Therefore, the direction of the hydrogen bond is preserved, with residue *i* donating the hydrogen bond to residue *i* − 2. Unlike the canonical β-turn, this structural feature does not reverse the direction of the polypeptide chain but creates kinks and turns of ∼110°.

#### 3.2.4. The C^δ1^—H → O=C (−3) class

**Figure 11:**
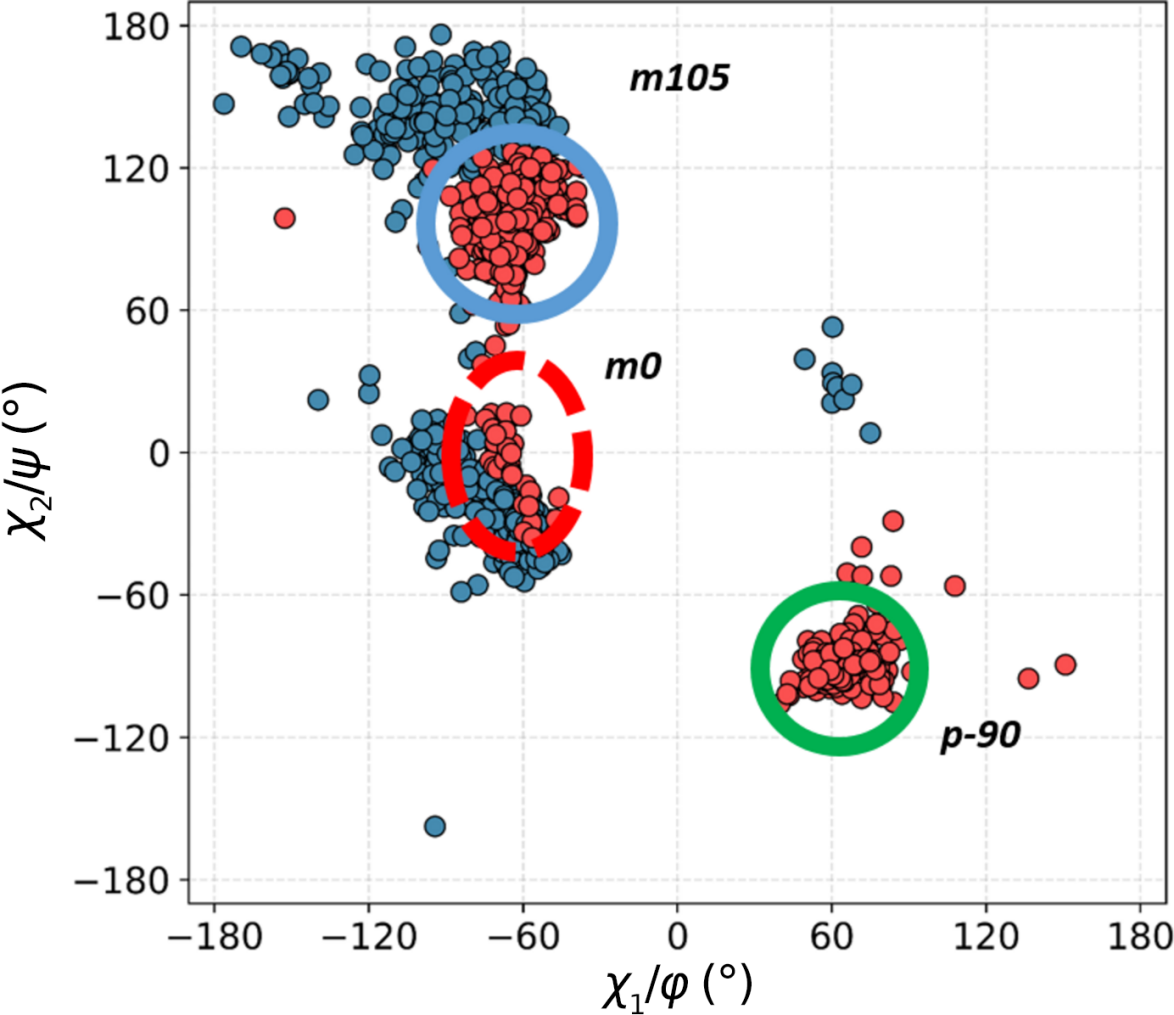
The −3 class of interactions. A double scatter plot (Ramachandran φ/ψ, blue; conformational, χ_1_/χ_2_, red) for Trp residues in all structural motifs in this class.

In this class, two amino acids are inserted between the acceptor carbonyl group and Trp, adding additional degrees of freedom. Nevertheless, we observe the presence of the same three conformational clusters as was the case for the −1 and −2 classes, *i.e.***m**105, **m**0 and **p**-90. The difference is that owing to weaker steric constraints, the **m**105 and **m**0 clusters are now distinctly separate and closer to the theoretical values for χ_2_ angles (averages of 98.5° and −3.6°, respectively), and the frequencies are decidedly shifted towards the canonical, low-energy conformations. There are 361 structures in the **m**105 cluster and 290 in the **p**-90 cluster, with only 34 in the unfavourable **m**0 group (Fig. 11 ▸).

The **m**105 cluster contains motifs with Trp found in both α and β secondary structures. The average α_H_ is 137.9°, but α_O_ is again unfavourable (average 113.8°). Except for a few outliers, the **p**-90 cluster is stereochemically tight, with a mean χ_1_ of 66° and χ_2_ of −89°. The vast majority of the motifs contain Trp in an α-helical form, and the putative hydrogen bond has a more favourable geometry, with an α_H_ of 138.5° and an α_O_ of 135.4°, with an average elevation of 0.6 Å on the *si* face. The small **m**0 cluster contains several motifs with Trp in α, β and left-handed helical secondary conformations. The *d*_HO_ distances are longer in this cluster, with an average α_H_ of 140.7° and α_O_ of 136.4°

**Figure 12:**
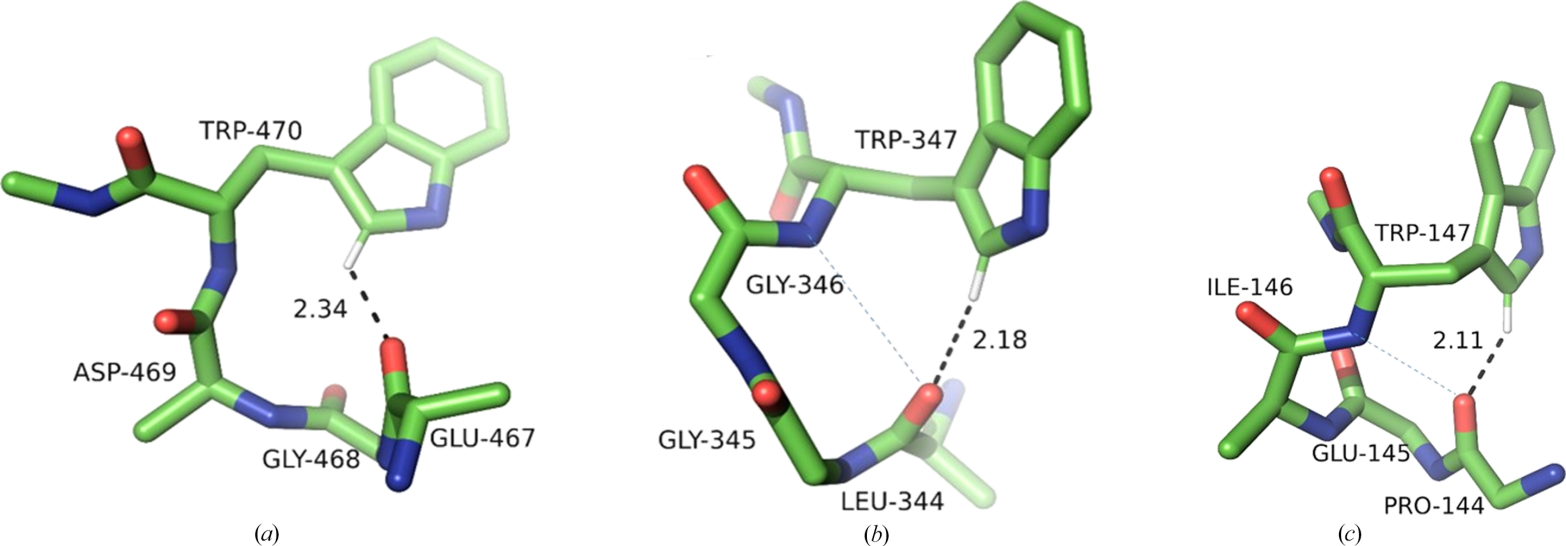
Examples of the three conformational Trp clusters in the +3 class. (*a*) **m**0 (PDB entry 5js4; only the C^β^ atoms of non-Trp residues are shown for clarity), (*b*) **m**105 (PDB entry 6x8o), (*c*) **p**-90 (PDB entry 6qo9). Note that (*b*) and (*c*) contain three-centred hydrogen bonds from the ^Trp^C^δ1^—H and amide groups to the *i* − 3 carbonyl reminiscent of a 3_10_-helical hydrogen-bonding pattern (canonical amide-to-carbonyl hydrogen bonds are shown as fine dashed lines).

Examples of a structural motif from each of the clusters are shown in Fig. 12 ▸.

#### 3.2.5. The C^δ1^—H → O=C (−4) class

**Figure 13:**
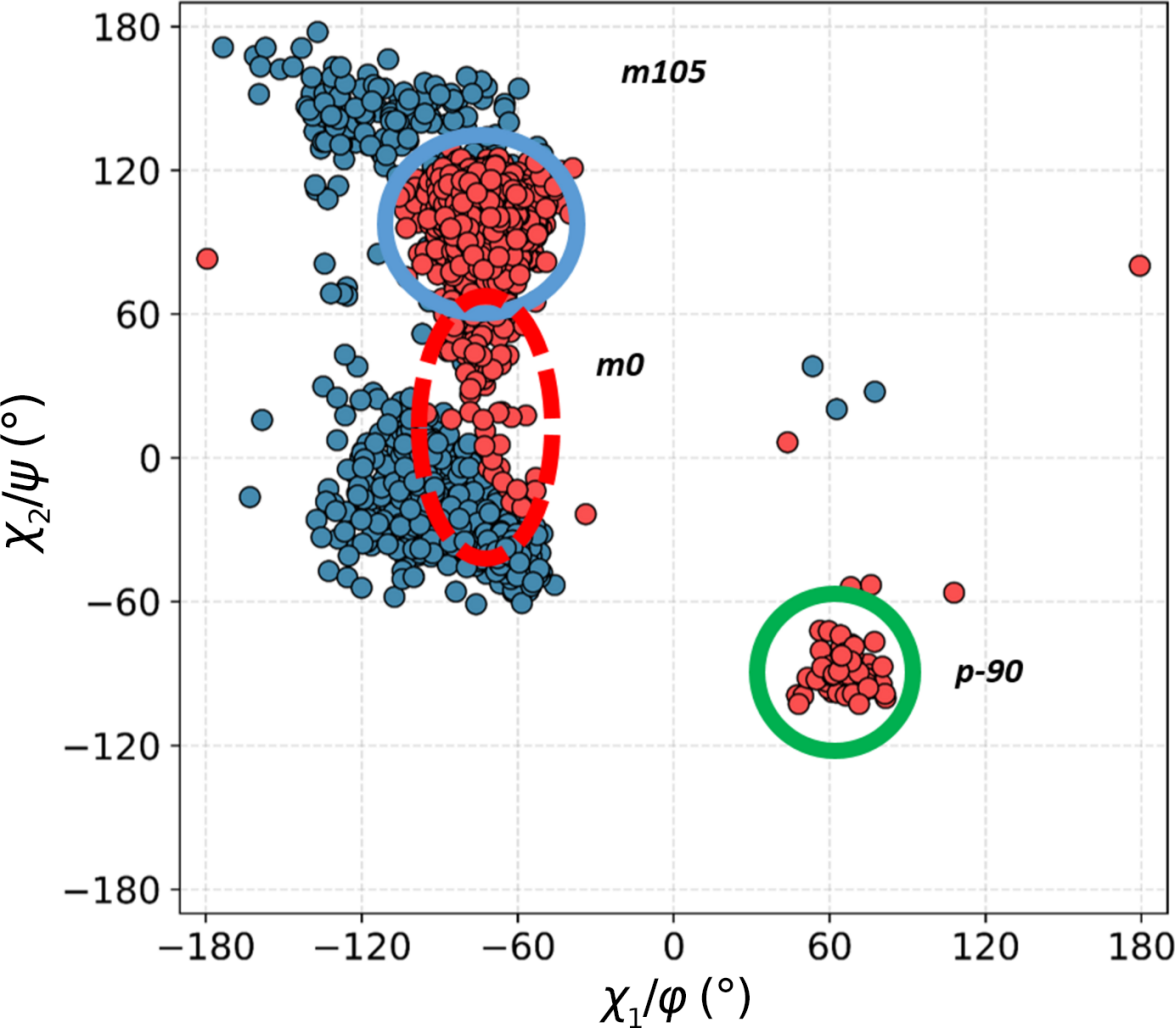
The −4 class of interactions. A double scatter plot (Ramachandran φ/ψ, blue; conformational, χ_1_/χ_2_, red) for Trp residues in all structural motifs in this class.

**Figure 14:**
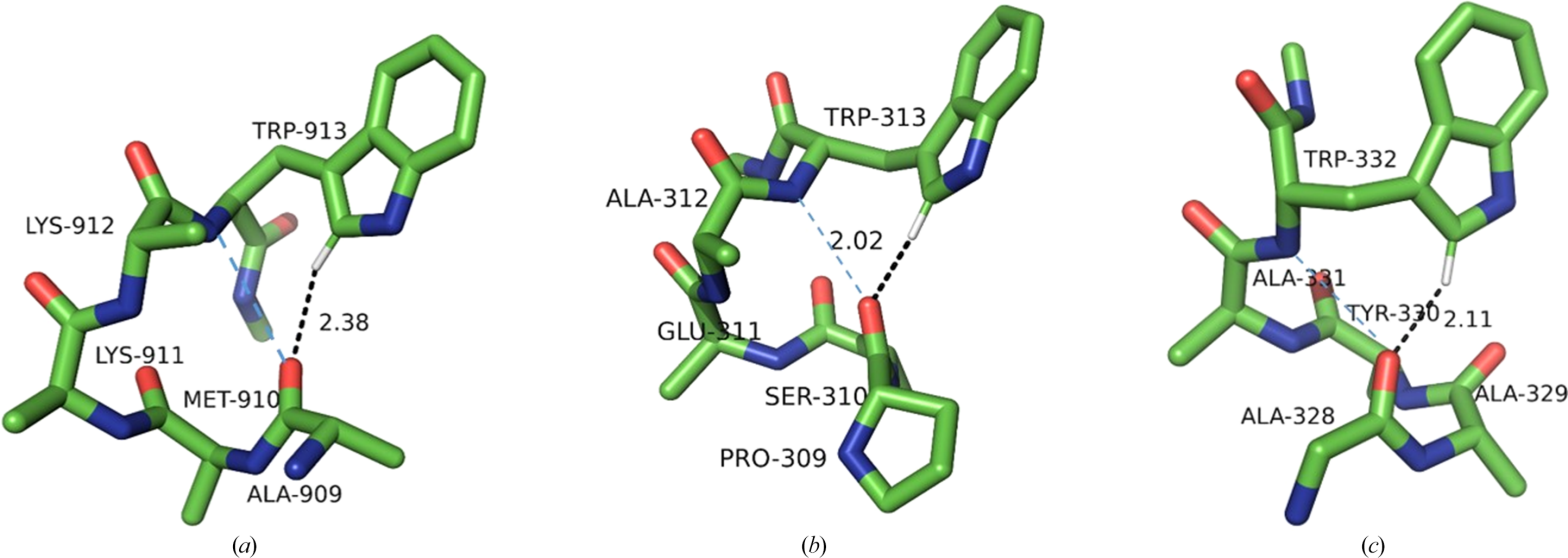
Examples of the three conformational Trp clusters in the +4 class. (*a*) **m**0 (PDB entry 7mzy; only the C^β^ atoms of non-Trp residues are shown for clarity, (*b*) **m**105 (PDB entry 4gxw), (*c*) **p**-90 (PDB entry 3s92). Note that all motifs contain hydrogen bonds from the ^Trp^C^δ1^—H and amide groups to the *i* − 4 carbonyl, capping it with a three-centred bond.

This is the most ubiquitous and the most diverse motif, owing to the flexibility generated by the insertion of three residues between the acceptor and donor amino acids. Nevertheless, perhaps surprisingly, only the same three conformational clusters are again present: **m**105, **m**0 and **p**-90. The canonical **m**105 conformer (average χ_2_ of 99°) is by far the most common, with nearly 1500 examples, compared with only 80 examples of **p**-90 and just 44 of **m**0 (Fig. 13 ▸). The majority, *i.e.* ∼75%, of motifs in the **m**105 cluster contain Trp in the α-helical conformation, often at the C-terminus of an α-helix (Fig. 14 ▸), capping the *i* − 4 carbonyl with three-centre hydrogen bonds donated by the main-chain amide and the C^δ1^—H group.

The 80 motifs in the **p**-90 cluster (average χ_2_ of −88°) contain primarily (85%) α-helical Trp, with a slightly more favourable average α_H_ of 138°. Most of these motifs also contain a three-centred hydrogen bond such that the amide group and C^δ1^—H cap the carbonyl O atom of residue *i* − 4. This is analogous to the recently documented capping of carbonyl O atoms within membrane helices by Thr and Ser hydroxyls, with a net gain of 127% in enthalpy compared with a single hydrogen bond (Brielle & Arkin, 2020 ▸).

The rare **m**0 motifs also contain Trp in both α and β secondary conformations. They tend to have an unfavourable angular stereochemistry, with an average α_H_ of 128° and α_O_ of 141°, and longer *d*_HO_ distances.

### 3.3. The interaction energies of C—H⋯O=C bonds

Whereas the stereochemical descriptors of close inter­atomic contacts provide useful information for the identification of hydrogen bonds, proximity *per se* does not imply a cohesive interaction or a structural function in the stabilization of a specific conformation. Historically, this was the argument used by Jerry Donohue in his criticism of June Sutor’s proposal for the existence of C—H⋯O bonds based on crystallographic data (Schwalbe, 2012 ▸). To support his view, he quoted Ramachandran’s opinion that H⋯O distances of 2.2 Å in proteins need not necessarily indicate the presence of a hydrogen bond (Ramachandran *et al.*, 1963 ▸). It is in principle true that the presence of a hydrogen bond is only hypothesized based on stereochemistry, and its strength is somewhat speculatively inferred from parameters such as linearity (α_H_) and hydrogen–acceptor distance (*d*_HO_). However, current knowledge of the physical chemistry of the hydrogen bond makes it possible to predict its existence based on the nature of the participating groups and stereochemistry with a very high degree of confidence. The nature of the various structural motifs described above, harbouring close C^δ1^—H⋯O=C interactions, is strongly suggestive of cohesive hydrogen bonds, but to assess the energies we turned to quantum-mechanical calculations.

It has been shown by one of us (Scheiner *et al.*, 2002 ▸) that a water molecule binds as a hydrogen-bond acceptor to the C^δ1^—H of indole with an energy of −2.1 kcal mol^−1^ at a *d*_CO_ distance of 3.35 Å. Because a peptide carbonyl is a stronger acceptor, we repeated this calculation for acetamide, representing an amide group, and indole as a model for Trp. The planes of the two molecules were perpendicular to avoid any steric repulsions, with a fully linear C—H⋯O=C arrangement. Following the optimization of *d*_HO_ (2.27 Å), we obtained a value for the energy of the interaction (*E*_int_) of −2.6 kcal mol^−1^, which is consistent with a stronger bond. (For comparison, we also calculated the *E*_int_ value for the inter­action of a carbonyl O atom of acetamide with the aromatic C^ɛ2^—H group of indole; the result was −1.05 kcal mol^−1^).

**Figure 15:**
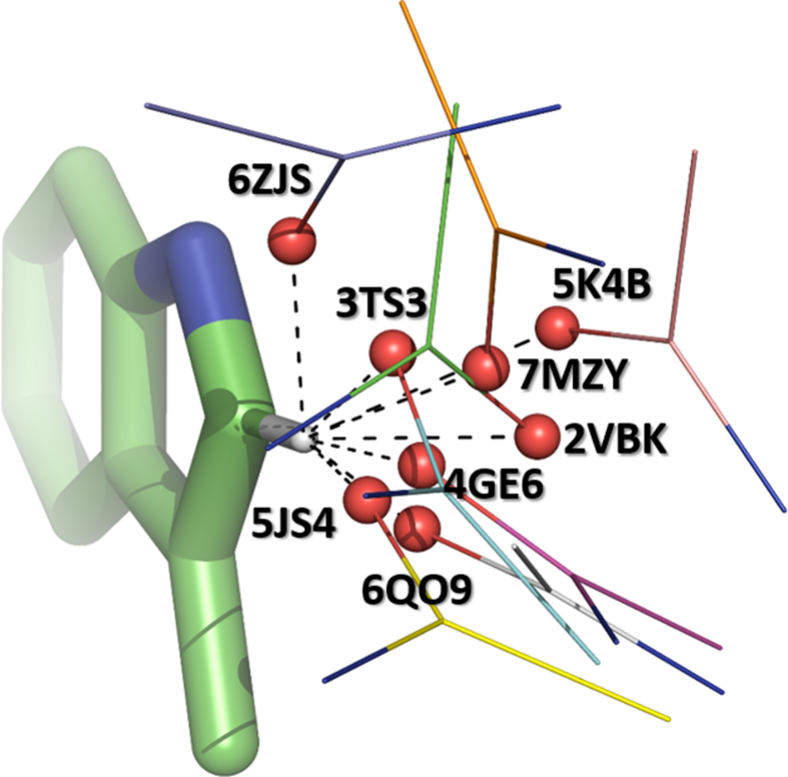
The stereochemistry of the C^δ1^—H⋯O=C interactions for which energies of interaction have been calculated (Table 1 ▸) superposed on the Trp side chain. The PDB codes are shown for each carbonyl O atom.

The above calculations use a perfectly linear C=O⋯H—C bond as a model system. The motifs found in actual protein structures are quite different from such ideal stereochemistry, and specifically many show α_H_ and α_O_ values that deviate significantly from linearity. We were interested in whether the energies of these interactions are still significant when compared with the reference system. To this end, we used eight representative cases from among those described above, with α_H_ ranging from 135° to 172°, α_O_ ranging from 94° to 165° and τ ranging from 0.3 to 2.15 Å. In each case, we truncated the Trp moiety to 3-methylindole and the acceptor peptide to *N*-methylacetamide, added H atoms using the *PyMOL* script and calculated interaction energies (*E*_int_; see Section 2). The results are shown in Table 1 ▸ and Fig. 15 ▸.

All interactions show cohesive *E*_int_ values irrespective of stereochemistry. As expected, the weakest *E*_int_ values were obtained for those interactions in which the H atom is located significantly out of the *sp*^2^ plane of the acceptor O atom. It appears that the α_H_ and α_O_ angles are less of a factor: both can be as low as ∼130° without a significant reduction in *E*_int_, as long as the hydrogen is within ∼0.8 Å of the *sp*^2^ plane.

We also noted that many of the structural motifs that we investigated show *d*_HO_ distances as short as ∼2.0 Å, significantly shorter than the predicted optimal distance of ∼2.3 Å. We wondered whether such short interactions, resulting from intramolecular constraints, might be less favourable.

**Figure 16:**
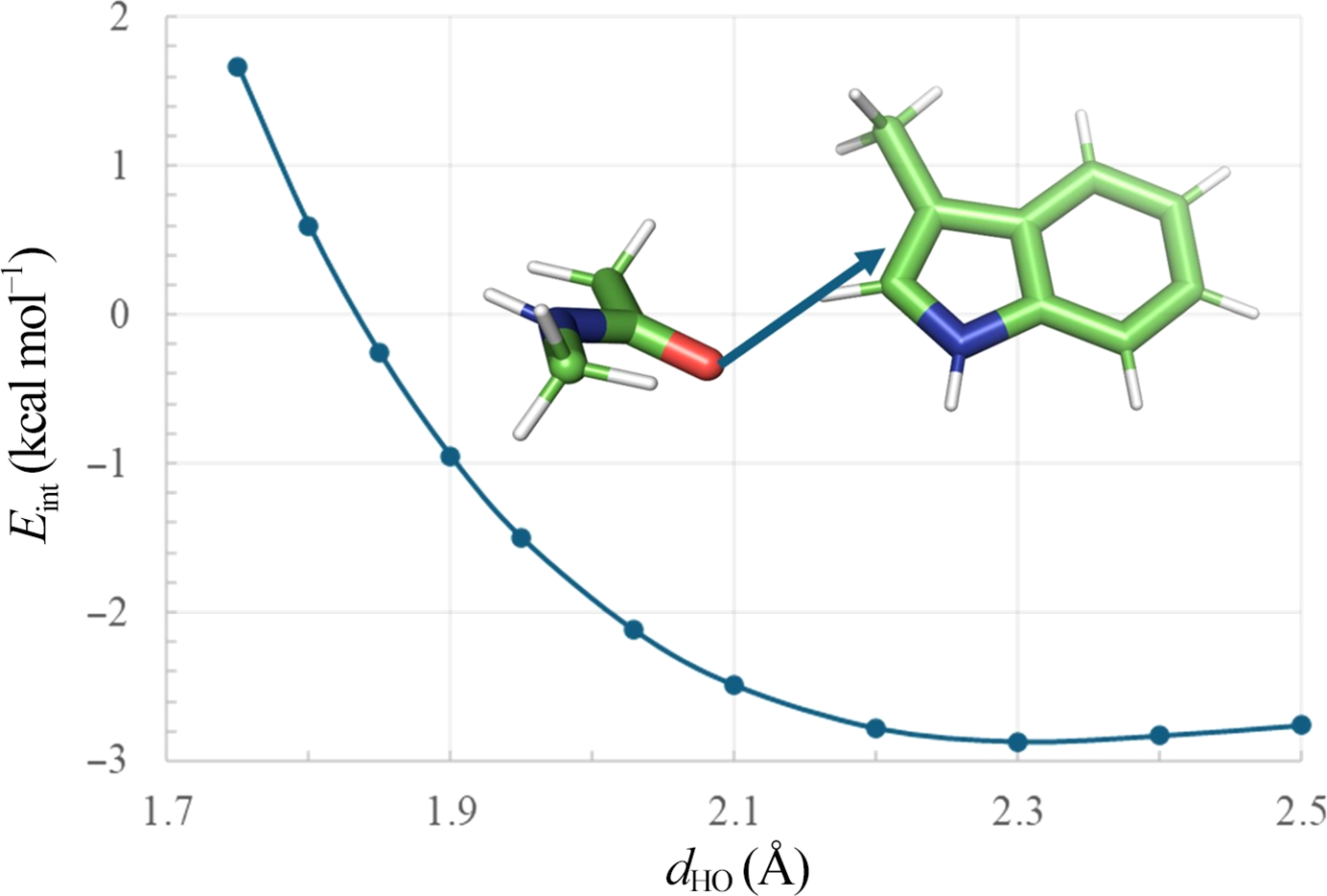
The dependence of the energy of interaction (*E*_int_) on the *d*_HO_ distance for an *N*-methylacetamide and 3-methylindole pair derived from PDB entry 3ts3. The arrow shows the position on the energy curve corresponding to the actual *d*_HO_ distance in the crystal structure, *i.e.* 2.02 Å. The black arrow indicates the line along which the 3-methylindole moiety was translated to obtain the curve of *E*_int_ versus distance.

We used PDB entry 3ts3 structure as a model case. We translated the 3-methylindole moiety along the H⋯O line and evaluated *E*_int_ between 2.5 and 1.8 Å (Fig. 16 ▸). We find that while *E*_int_ reaches a maximum at 2.3 Å, the interaction is cohesive down to ∼1.85 Å, which corresponds well to the shortest observed contacts in the crystal structures. There is little loss of energy when the bond is stretched to 2.5 Å, consistent with the primarily electrostatic nature of the interaction.

## 4. Conclusions

It is well established that main-chain/side-chain interactions mediated by hydrogen bonds are involved in specific conformational motifs, often capping secondary-structure elements such as helices and β-sheets (Eswar & Ramakrishnan, 2000 ▸; Krishna Deepak & Sankararamakrishnan, 2016 ▸). However, such motifs reported to date invariably involved canonical hydrogen bonds, *i.e.* those involving N and O atoms. Typical examples are Asx-turns, in which the side-chain carbonyl O atom of Asp or Asn engages the main-chain amide of the *i* + 2 residue, mimicking a β-turn (D’mello *et al.*, 2022 ▸). Similarly, N^δ1^ of the histidine imidazole has been shown to engage with the backbone amide groups (Krishna Deepak & Sankararamakrishnan, 2016 ▸). Interestingly, C^δ1^ of Trp occupies a position isosteric to O^δ^ of Asx and N^δ1^ of His, and because it is protonated it engages the carbonyl and not the amide groups of the main chain. Our study demonstrates that the C^δ1^—H group of a tryptophan residue plays an important role in stabilizing unique structural motifs by engaging as an hydrogen-bond donor with main-chain carbonyl O atoms nearby in the sequence. The most common such interactions involve residues one peptide unit downstream, *i.e.i* +1, or 1–4 peptide units upstream, *i.e.i* − 1 to *i* − 4. Interestingly, Trp is found in these motifs in only six of the possible nine conformers, with the *i* + 1 class containing only **p**90, **t**0 and **t**-105 conformers, while the remaining four classes show Trp only in **m**105, **m**0 and **p**-90 conformations. The frequencies of the high-energy **m**0 and **t**0 conformers is increased significantly in those classes where the contacts are strongly restricted by short-range steric constraints, while **m**105, the most populous class found in proteins, is strongly enriched in the −3 and −4 classes. Our work helps to explain the relatively common occurrence of the **m**0 and **t**0 classes. It is important to note that the function of Trp residues is intimately contingent on their conformation. For example, Trp in transmembrane helices occurs most often in **m**0, **t**0 and **p**-90 conformations, all of which have been characterized in our study (de Jesus & Allen, 2013 ▸). Of importance is our observation that in the −3 and −4 classes Trp is often engaged in capping the acceptor O atom with hydrogen bonds donated by both the amide and C^δ1^—H groups.

We also present evidence based on quantum-chemical calculations that the short C^δ1^—H⋯O=C contacts revealed by structural data mining are in fact invariably cohesive interactions of the order of approximately half a canonical hydrogen bond, and less sensitive to specific stereochemistry, such as C—H⋯O and H⋯O=C angles, than previously thought. The critical factor is the position of the H atom close to the *sp*^2^ plane of the acceptor O atom.

## 5. Related literature

The following references are cited in the supporting information for this article: Adams *et al.* (2010 ▸), Emsley *et al.* (2010 ▸) and Kovalevskiy *et al.* (2018 ▸).

## Supplementary Material

Note on the precision of the crystallographic coordinates and Supplementary Methods. DOI: 10.1107/S2059798324005515/chr5002sup1.pdf

Supplementary Table S1. Crystallographic re-refinement of structural models analysed in the paper. DOI: 10.1107/S2059798324005515/chr5002sup2.xlsx

## Figures and Tables

**Figure 3:**
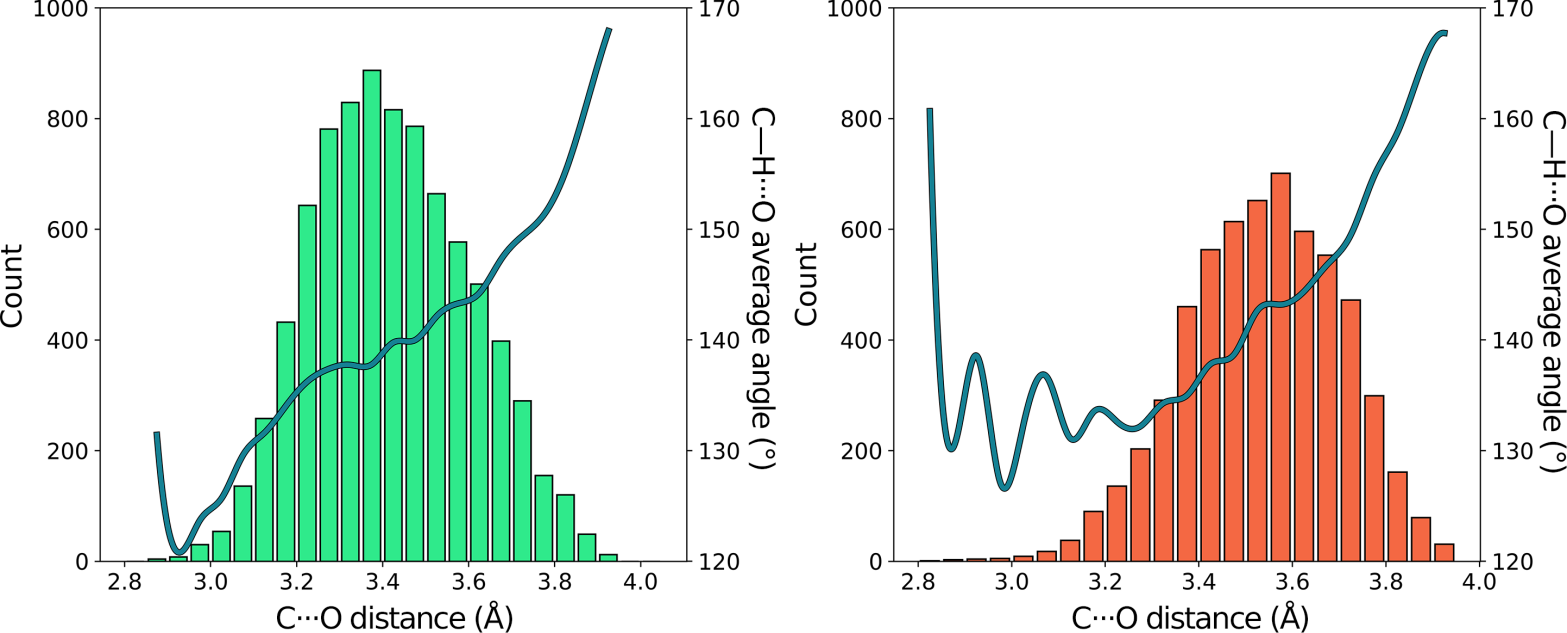
Left: a histogram of the number of interactions of ^Trp^C^δ1^—H with backbone carbonyl O atoms as a function of the distance *d*_CO_ (green bars) and a mean value of the α_H_ angle in each group, corrected with cubic interpolation. Right: the same statistics for interactions with water molecules.

**Table 1:** Interaction energies (*E*_int_) calculated for 3-methylindole and *N*-methylacetamide pairs based on the coordinates of specific interactions in protein structures The Δ*d*_CO_ values are the changes in the C⋯O distance (*d*_CO_) resulting from additional refinement; the final *d*_HA_ values (*i.e.* hydrogen⋯acceptor distances) were obtained after the riding H atoms were replaced with those calculated by *PyMOL*. Other parameters are α_H_ (the C—H⋯O angle), α_O_ (the C=O⋯H angle) and τ (the elevation of H from the *sp*^2^ plane).

PDB entry	Class	Conformer	*d*_CO_ (Å)	Δ*d*_CO_ (Å)	α_H_ (°)	α_O_ (°)	τ (Å)	*d*_HA_ (Å)	Trp	Acceptor	*E*_int_ (kcal mol^−1^)
3ts3	1	**t**0	3.026	0.027	153	120	1.36	2.029	612	Ser613	−2.12
4ge6	1	**t**-105	3.143	0.052	172	128	0.61	2.109	468B	Pro469	−2.84
6qo9	−2	**p**-90	3.098	0.005	171	162	0.28	2.023	26B	Gln24B	−2.81
5k4b	−2	**m**0	2.862	0.069	140	153	−0.76	2.107	419A	Val417	−1.57
2vbk	−2	**m**105	3.245	0.025	160	94	−2.15	2.230	304A	Gly302	−1.53
6zjs	−3	**m**0	3.167	0.018	135	165	−0.42	2.343	470A	Glu467	−2.94
5js4	−3	**m**105	3.245	−0.023	166	152	0.80	2.183	347A	Leu344	−2.74
7mzy	−4	**m**0	3.405	0.017	163	137	−0.52	2.375	913A	Ala909	−2.41
