# Supplementary material for: A structural role for tryptophan in proteins, and the ubiquitous Trp Cδ1—H⋯O=C (backbone) hydrogen bond
Source: Acta Crystallogr D Struct Biol. 2024 Jun 28;80(Pt 7):551–62. doi: 10.1107/S2059798324005515 (PMC11220837; doi:10.1107/S2059798324005515)
Supplement: Supplementary file 1 [file d-80-00551-sup1.pdf]

STRUCTURAL BIOLOGY

Volume 80 (2024)

Supporting information for article:

A structural role for tryptophan in proteins, and the ubiquitous Trp $C^{\delta 1}$—H···O=C (backbone) hydrogen bond

Michal Szczygiel, Urszula Derewenda, Steve Scheiner, Wladek Minor and Zygmunt S. Derewenda

S1. Note on the precision of the crystallographic coordinates

The PDB does not enforce any specific protocols or metrics, and as a result, most sets of coordinates are not the most precise—given the available data—but rather represent somewhat arbitrary endpoints decided upon by the investigator. We were specifically concerned about lack on inclusion of hydrogen atoms in the vast majority of cases, regardless of resolution, and premature anisotropic refinement carried in the absence of hydrogen atoms. Therefore, for each of the structures, we inspected the electron density maps to ensure that the Trp was well resolved and correctly positioned in the density, and we re-refined all models using diffraction data deposited in the PDB (see Methods for refinement protocol). Table S1 shows the results of our efforts. We were correct in assuming that the coordinate sets often lack precision. Re-refinement resulted in most cases in reduced R and R-free factors, and in the majority of structures, the geometry of the models has improved, as judged by r.m.s. deviations from target values of bonds and angles. Importantly, the distances between the relevant heavy atoms in hydrogen bonds, i.e. carbon and oxygen, changed during the refinement, for those structures were hydrogen atoms were not included in the original models, increasing by as much as 0.075 Å. This is because with hydrogens absent in the models refined at high resolution, the positions of the centres of gravity of electron density shift slightly towards the hydrogen to compensate for their lack. However, in those models that were originally refined specifically by REFMAC(Kovalevskiy *et al.*, 2018) using riding hydrogen atoms, we observed some shortening of distances, likely owing to different sets of geometric constraints. This is a cautionary tale for computational chemists starting from raw crystallographic coordinates. All specific stereochemical measurements for select cited henceforth are based on re-refined coordinates; average values for whole clusters are based on raw models.

S2. Supporting Methods: Crystallographic Refinement Protocol.

All crystal structures used for detailed analysis as representative motifs were first inspected using the graphical program COOT (Emsley *et al.*, 2010) to assess the quality of the electron density map. Only structures for which diffraction data was available from the PDB were used at this point. Cases of Trp residues exposed to solvent with higher than average atomic displacement parameters (ADPs) were not studies further (also they are included in the general databases). Any structures with obvious gross errors that might affect the overall precision of coordinates were also rejected. Structures selected in this way for further analysis were subjected to standardized crystallographic refinement that incorporated both riding-hydrogens and anisotropic atomic displacement factors refinement. The program PHENIX (Adams *et al.*, 2010) was used for this purpose. The first round consisted of three cycles of refinement of non-hydrogen atoms, with isotropic ADPs reset to 10 $Å^2$. Solvent was updated in each cycle. The second round of 5 cycles involved addition of riding hydrogens, followed by round 3 in which anisotropic ADPs were introduced. As PHENIX add riding hydrogens at the incorrect

distance of 0.93 Å, we recalculated the positions using PYMOL, and the final sets of coordinates had the $C_{\delta1}$-H distance reset to 1.09 Å. Table S1 contains the results.
